# Respiratory Physiology of Lactococcus lactis in Chemostat Cultures and Its Effect on Cellular Robustness in Frozen and Freeze-Dried Starter Cultures

**DOI:** 10.1128/AEM.02785-19

**Published:** 2020-03-02

**Authors:** Anna Johanson, Anisha Goel, Lisbeth Olsson, Carl Johan Franzén

**Affiliations:** a Division of Industrial Biotechnology, Department of Biology and Biological Engineering, Chalmers University of Technology, Gothenburg, Sweden; b Chr. Hansen A/S, Hørsholm, Denmark; The Pennsylvania State University

**Keywords:** lactic acid bacteria, continuous cultures, respiration-permissive conditions, acidification activity, aerobic growth, microbial physiology

## Abstract

In this study, we used chemostat cultures to analyze the quantitative effects of the specific growth rate and respiration on the metabolism in Lactococcus lactis CHCC2862 and on the downstream robustness of cells after freezing or freeze-drying. Under anaerobic conditions, metabolism remained homofermentative, although biomass yields varied with the dilution rate (*D*). In contrast, metabolism shifted with the dilution rate under respiration-permissive conditions. At *D* = 0.1 h^−1^, no lactate was produced, while lactate formation increased with higher dilution rates. Thus, a clear metabolic shift was observed, from flavor-forming respiratory metabolism at low specific growth rates to mixed-acid respiro-fermentative metabolism at higher specific growth rates. Quantitative analysis of the respiratory activity, lactose uptake rate, and metabolite production rates showed that aerobic acetoin formation provided most of the NADH consumed in respiration. Moreover, the maintenance-associated lactose consumption under respiration-permissive conditions was only 10% of the anaerobic value, either due to higher respiratory yield of ATP on consumed lactose or due to lower maintenance-related ATP demand. The cultivation conditions also affected the quality of the starter cultures produced. Cells harvested under respiration-permissive conditions at *D* = 0.1 h^−1^ were less robust after freeze-drying and had lower acidification activity for subsequent milk acidification, whereas respiration-permissive conditions at the higher dilution rates led to robust cells that performed equally well or better than anaerobic cells.

**IMPORTANCE** Lactococcus lactis is used in large quantities by the food and biotechnology industries. L. lactis can use oxygen for respiration if heme is supplied in the growth medium. This has been extensively studied in batch cultures using various mutants, but quantitative studies of how the cell growth affects respiratory metabolism, energetics, and cell quality are surprisingly scarce. Our results demonstrate that the respiratory metabolism of L. lactis is remarkably flexible and can be modulated by controlling the specific growth rate. We also link the physiological state of cells during cultivation to the quality of frozen or freeze-dried cells, which is relevant to the industry that may lack understanding of such relationships. This study extends our knowledge of respiratory metabolism in L. lactis and its impact on frozen and freeze-dried starter culture products, and it illustrates the influence of cultivation conditions and microbial physiology on the quality of starter cultures.

## Abstract (precis)

Lactococcus lactis is used in large quantities by the food and biotechnology industries. L. lactis can use oxygen for respiration if heme is supplied in the growth medium. This has been extensively studied in batch cultures using various mutants, but quantitative studies of how the cell growth affects respiratory metabolism, energetics, and cell quality are surprisingly scarce. Our results demonstrate that the respiratory metabolism of L. lactis is remarkably flexible and can be modulated by controlling the specific growth rate. We also link the physiological state of cells during cultivation to the quality of frozen or freeze-dried cells, which is relevant to the industry that may lack understanding of such relationships. This study extends our knowledge of respiratory metabolism in L. lactis and its impact on frozen and freeze-dried starter culture products, and it illustrates the influence of cultivation conditions and microbial physiology on the quality of starter cultures.

## INTRODUCTION

Lactococcus lactis is extensively used within the dairy industry as a starter culture. Starter cultures are produced in a process involving cultivation, concentration, and preservation, for example, by freezing or freeze-drying (1). Lactic acid bacteria are commonly freeze-dried, as this ensures not only long-term preservation, but also lower storage and transportation costs than for frozen cell suspensions. Bacterial tolerance to freeze-drying varies among strains and can have considerable impact on the metabolic activity, e.g., acidification rate, in the final application. Moreover, the robustness of the starter culture, *viz*., cell survival and metabolic capacity after storage, is also affected by the conditions under which it is produced, e.g., cultivation conditions and exposure to cold and heat shock (2, 3). The industry strives to achieve starter culture production conditions that give high biomass yield without compromising cellular robustness.

L. lactis has long been described as an anaerobic homofermentative bacterium which converts at least 90% of consumed sugars to lactate. However, it is now known that L. lactis can undergo aerobic respiration in the presence of heme and oxygen, so-called respiration-permissive conditions (4–7). Heme is an essential cofactor for the cytochrome oxidase system. In the presence of exogenous heme, L. lactis can establish an electron transport chain (ETC) consisting of NADH dehydrogenase, menaquinone as an electron shuttle, and cytochrome oxidase, resulting in NADH oxidation and proton extrusion from the cell (6, 8–10).

**FIG 1:**
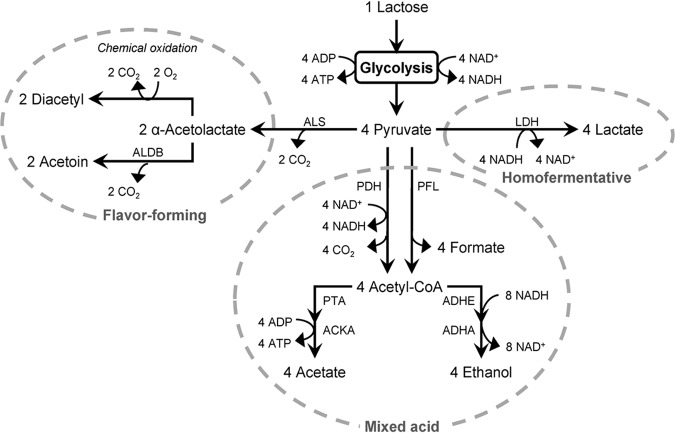
Homofermentative, mixed acid and flavor-forming metabolism of lactose in L. lactis. LDH, lactate dehydrogenase; PFL, pyruvate formate lyase; PDH, pyruvate dehydrogenase; ADHE, acetaldehyde dehydrogenase; ADHA, alcohol dehydrogenase; PTA, phosphotransacetylase; ACKA, acetate kinase; ALS, acetolactate synthase; ALDB, acetolactate decarboxylase. Stoichiometries assume complete degradation of lactose into each metabolite. Adapted from references 40 and 41.

L. lactis strains are generally considered to be homofermentative under anaerobic conditions, where pyruvate is reduced to lactate via lactate dehydrogenase (LDH) (Fig. 1). Conditions such as carbon limitation and low specific growth rate can result in a decrease in the flux through LDH. Under such conditions, pyruvate is instead metabolized via pyruvate formate lyase (PFL) or pyruvate dehydrogenase (PDH), leading to mixed-acid fermentation, including production of formate, CO_2_, acetate, and ethanol in addition to lactate (11–14). The significant difference between PDH and PFL is that PDH produces NADH and CO_2_, rather than formate, in addition to acetyl-CoA. Furthermore, PDH activity is induced by aeration, whereas PFL is inactivated. Irrespective of whether PDH or PFL is activated, the intermediate acetyl-CoA is further metabolized to either acetate or ethanol, via phosphotransacetylase (PTA) and acetate kinase (ACKA), or via acetaldehyde dehydrogenase (ADHE) and alcohol dehydrogenase (ADHA), respectively. Acetate formation generates additional ATP, while ethanol formation regenerates NAD^+^.

During anaerobic fermentation, when lactate is the main product, protons are extruded by the H^+^-ATPase to maintain the intracellular pH at the expense of ATP (8, 15). H^+^-ATPase has been recognized as being essential for the anaerobic growth of L. lactis (16). However, in the presence of heme, the ETC functions as a proton transport system causing respiration-driven efflux of protons in addition to, or instead of, H^+^-ATPase (17). L. lactis thus saves ATP through heme-activated respiration, as NADH dehydrogenase regenerates NAD^+^ while protons are extruded at no additional ATP cost. Some studies suggest that additional ATP generation may occur; however, this has not yet been fully verified (8, 15).

Furthermore, respiration-permissive conditions direct metabolism toward flavor-forming pathways. The flux from pyruvate to lactate decreases significantly, and acetoin, diacetyl, and CO_2_ are formed instead. In the production of acetoin and diacetyl, pyruvate undergoes decarboxylation by acetolactate synthase (ALS) into acetolactate (Fig. 1). Acetolactate is unstable and can either undergo spontaneous conversion to diacetyl by a chemical reaction with O_2_, or it can be enzymatically converted to acetoin by acetolactate decarboxylase (18).

The advantages of respiration-permissive conditions are well established in both the literature and in industrial practice; the intracellular oxygen level is decreased, acid production is reduced, and thus, the stress on the cells is lower. This leads to an increase in biomass yield, resistance to oxygen, and long-term cellular survival in liquid-form cultures stored at 4°C (4, 5, 19). Nevertheless, respiration in L. lactis has exclusively been conducted in batch cultivations and at low sugar concentrations. Quantitative analyses of metabolic fluxes and energetics are scarce and have considered neither the potential relationship between the specific growth rate and the beneficial effects of respiration nor the potential effects on the robustness in terms of tolerance to downstream processing.

The aim of the present study was to understand the relationship between respiration-permissive conditions during starter culture production and the microbial robustness during downstream treatment of the culture. We mimicked conditions that prevail during different phases of industrial batch production by culturing L. lactis subsp. *lactis* to steady states at three dilution rates in respiration-permissive and anaerobic chemostat cultures. We performed quantitative metabolic analysis to investigate how cellular metabolism and energetics depend on respiration and the specific growth rate. Furthermore, we explored how the specific growth rate and respiration influence the robustness of frozen and freeze-dried starter cultures by assessing the viability and acidification activity after industry-like downstream processing.

## RESULTS

### Metabolic fluxes and yields.

**FIG 2:**
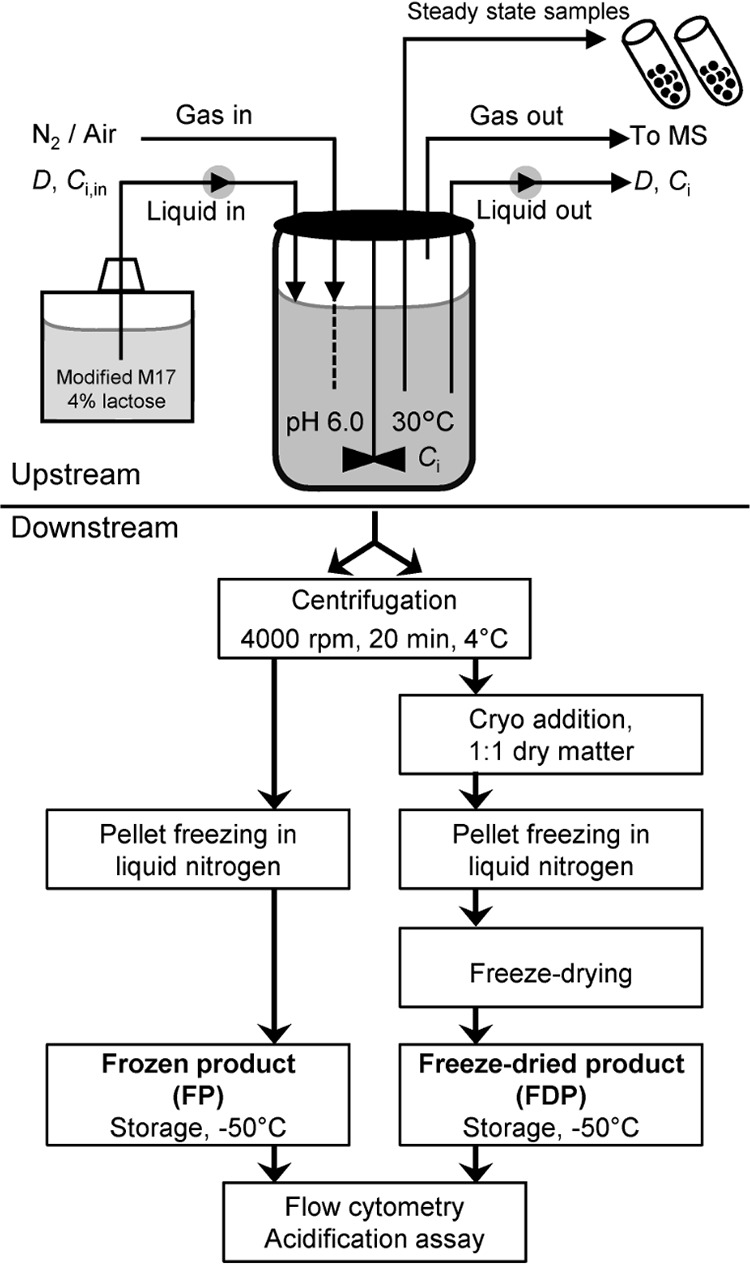
Overview of experimental procedures, showing the upstream and downstream processes included in this study: fermentation, separation, freezing, and/or freeze-drying. For each fermentation condition, cells were collected after centrifugation of the fermentation broth (FB), after freezing in liquid nitrogen (FP), and after freeze-drying (FDP). For anaerobic operation, the headspace was flushed with N_2_. For aerobic conditions, air was sparged under the impeller. Steady state was obtained at dilution rates (*D*) of 0.1 h^−1^, 0.5 h^−1^, and 0.8 h^−1^ from individual startup cultures. Samples were withdrawn directly from the bioreactor and quenched on cold stainless-steel balls.

Lactococcus lactis CHCC2862 was grown in continuous cultures on rich medium with 40 g liter^−1^ lactose under respiration-permissive conditions, i.e., aerobically and with hemin supplementation, and under anaerobic conditions in order to study the effects of respiration and specific growth rate on metabolite yields, energetics of growth, and robustness during and after downstream processing. The experimental procedures are schematically illustrated in Fig. 2.

**FIG 3:**
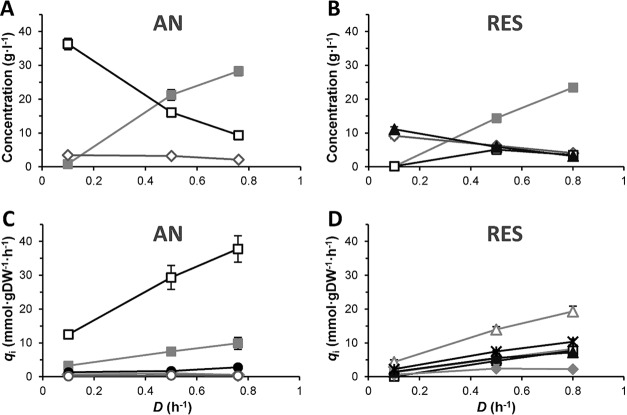
Overview of concentrations and rates of consumption and production of main substrates, products, and biomass in chemostat cultivations. (A and C) Anaerobic conditions; (B and D) respiration-permissive conditions. (A and B) Concentrations; (C and D) specific consumption and production rates determined at steady state. Symbols: gray squares, lactose; white squares, lactate; white diamonds, biomass; black triangles, acetoin; gray diamonds, acetate; black circles, formate; white circles, ethanol; white triangles, CO_2_; black stars, O_2_. Data points represent the average of biological duplicates, and error bars, the 95% confidence interval from measurements of two separate steady-state samples of each cultivation.

The residual lactose concentration increased with increasing dilution rates under both anaerobic and respiration-permissive conditions (Fig. 3A and B). The biomass concentration was higher under respiration-permissive than anaerobic conditions. Lactate was the main product under anaerobic conditions (Fig. 3A and C), whereas acetoin was the main product under respiration-permissive conditions, together with CO_2_ and lactate, depending on the dilution rate (Fig. 3B and D, Table 1). At *D* = 0.1 h^−1^, no detectable lactate was formed in the respiration-permissive chemostat.

**TABLE 1:** Metabolite yields (C-mol C-mol^−1^) on lactose under anaerobic conditions (AN) and respiration-permissive (RES) conditions^*a*^

Dilution rate	Yield on lactose (C-mol C-mol^−1^)									C recovery excluding biomass (%)
	Biomass	Lactate	Acetate	Acetoin	Diacetyl	Ethanol	Formate	CO_2_	O_2_	
AN 0.1 h^−1^	0.11 ± 0.01	0.96 ± 0.03	0.03 ± 0.00	ND	ND	0.01 ± 0.00	0.03 ± 0.00	TA	ND	104
AN 0.5 h^−1^	0.24 ± 0.03 (*)	0.99 ± 0.13	0.02 ± 0.00 (*)	ND	ND	0.01 ± 0.00 (*)	0.02 ± 0.00 (*)	TA	ND	104
AN 0.8 h^−1^	0.26 ± 0.04 (*, –)	0.96 ± 0.09	0.01 ± 0.00 (*, *)	ND	ND	0.00 ± 0.00 (*, *)	0.02 ± 0.00 (*, *)	TA	ND	100
RES 0.1 h^−1^	0.29 ± 0.03	0.00 ± 0.00	0.07 ± 0.00	0.39 ± 0.03	0.01 ± 0.00	TA	ND	0.30 ± 0.05	0.15 ± 0.02	78
RES 0.5 h^−1^	0.32 ± 0.03	0.22 ± 0.01 (*)	0.07 ± 0.00	0.34 ± 0.03	0.01 ± 0.00	TA	ND	0.22 ± 0.02 (*)	0.12 ± 0.00 (*)	87
RES 0.8 h^−1^	0.33 ± 0.04	0.23 ± 0.02 (*, –)	0.05 ± 0.00 (*, *)	0.29 ± 0.04 (*, *)	0.01 ± 0.00	TA	ND	0.20 ± 0.02 (*, –)	0.11 ± 0.01 (*, –)	78

a Values given are the average of biological duplicates and the 95% confidence interval from measurements of two separate steady-state samples of each cultivation. The cell biomass was not included in the carbon recovery (C recovery) calculation. (TA, trace amount; ND, not detected). Data in parentheses indicate significance levels at *P* > 0.05 (–) and *P* < 0.05 (*) of the yields compared between dilution rates for anaerobic and respiration-permissive conditions. For *D* = 0.5 h^−1^, the entry indicates the statistical significance compared to *D* = 0.1 h^−1^. For *D* = 0.8 h^−1^, the first entry indicates the statistical significance compared to *D* = 0.1 h^−1^, and the second, compared to *D* = 0.5 h^−1^. At each dilution rate, all metabolite and biomass yields were significantly different between anaerobic and respiration-permissive conditions with *P* < 0.05.

Under anaerobic conditions, lactose was almost exclusively converted to lactate, and only minor amounts of formate, acetate, and ethanol were formed (Fig. 3C, Table 1). The metabolism remained homofermentative, with mainly lactate being produced at all dilution rates. Under respiration-permissive conditions, the metabolite profile instead consisted of a mixture of acetoin, CO_2_, acetate, and lactate, with CO_2_ exhibiting the highest specific flux (Fig. 3D, Table 1). At *D* = 0.1 h^−1^ the lactate flux was close to zero, but it increased linearly with increasing dilution rates. The specific O_2_ uptake rate also increased with increasing dilution rates (Fig. 3D; *P* < 0.05). However, the ratio between CO_2_ production and O_2_ uptake (the respiratory quotient, *RQ*) was higher at *D* = 0.1 h^−1^ and *RQ* = 1.97 than at *D* = 0.5 h^−1^ and 0.8 h^−1^ where *RQ* = 1.88 (*P* < 0.05). This was in line with the higher activity of CO_2_-producing metabolic pathways at *D* = 0.1 h^−1^, where acetoin was the main product (Table 1). Diacetyl was detected in all respiratory cultures but at low concentrations.

Under anaerobic conditions, the biomass yield on consumed lactose was significantly lower (*P* < 0.05) at *D* = 0.1 h^−1^ (0.11 mol carbon [C-mol] C-mol^−1^) than at *D* = 0.5 h^−1^ and *D* = 0.8 h^−1^ (0.24 and 0.26 C-mol C-mol^−1^, respectively). Lactate was the main product, and lactate yields were all similar at 0.96 to 0.99 C-mol C-mol^−1^ (Table 1). Acetate yields were low and decreased significantly with increasing dilution rate (*P* < 0.05).

Under respiration-permissive conditions, the biomass yield at *D* = 0.1 h^−1^ tended to be lower than that at *D* = 0.5 h^−1^ and *D* = 0.8 h^−1^; however, the differences were much smaller. At *D* = 0.1 h^−1^, no lactate was produced, and carbon was instead directed to the flavor-forming and mixed acid metabolites acetoin, acetate, and CO_2_. The lactate yield on consumed lactose increased with increasing dilution rates, while acetoin and CO_2_ yields decreased (Table 1; *P* < 0.05).

### Energy and redox balances.

**FIG 4:**
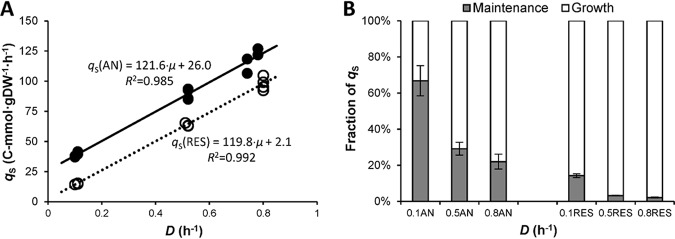
Determination of substrate-related maintenance. (A) Plot of the specific lactose consumption (*q*_S_, C-mmol gDW^−1^ h^−1^) versus the dilution rate (*D*, h^−1^) to calculate the substrate-related maintenance coefficient and maximum biomass yield. Filled circles, anaerobic (AN); open circles, respiration-permissive (RES) conditions. (B) Distribution (C-mmol C-mmol^−1^) of specific lactose consumption rate between maintenance-related (gray) and growth-related (white) processes under anaerobic (AN) and respiration-permissive (RES) conditions. The error bars represent the 95% confidence interval.

The substrate-related maintenance coefficient (*m*_S_) and maximum biomass yield on lactose (*Y*_SX_^max^) of L. lactis CHCC2862 were estimated from the specific lactose consumption by linear regression. The relationships between the specific lactose consumption rate and the dilution rate were linear and clearly significant under both anaerobic and respiration-permissive conditions (observed *F*-test statistic *F*_obs, AN_ = 656 > *F*_0.001,1,10_ = 21 and *F*_obs, RES_ = 1,072 > *F*_0.001,1,9_ = 23, respectively), indicating that the maintenance coefficient was independent of the specific growth rate in both cases (Fig. 4A). The maintenance coefficient was significantly lower under respiration-permissive conditions, 2.1 ± 4.7 mmol of carbon per gram dry cell weight per hour (C-mmol gDW^−1^ h^−1^, 95% confidence interval) than under anaerobic conditions (26.0 ± 5.6 C-mmol gDW^−1^ h^−1^). The maximum biomass yields on lactose were very similar under respiration-permissive and anaerobic conditions, 1/(120 ± 8) and 1/(122 ± 10) gDW C-mmol^−1^, respectively, corresponding to 0.33 ± 0.03 C-mol C-mol^−1^.

As a consequence, the fraction of carbon (C-mol C-mol^−1^) used for maintenance under respiration-permissive conditions was 14%, 3%, and 2% of the consumed lactose at *D* = 0.1 h^−1^, 0.5 h^−1^, and 0.8 h^−1^, respectively (Fig. 4B). The maintenance energy demand was higher under anaerobic conditions; 67%, 29%, and 22% of the consumed lactose was used for maintenance at *D* = 0.1 h^−1^, 0.5 h^−1^, and 0.8 h^−1^, respectively.

The NADH-NAD^+^ balance in the central carbon metabolism and the ATP produced via substrate-level phosphorylation, *ATP*_SLP_, were estimated based on the lactose catabolism and yields of all by-products (Table 2). Under anaerobic conditions, the balance of NADH formed and NAD^+^ regenerated in catabolism (see equation 2) was almost complete, indicating small experimental errors. An excess of NADH was found in lactose catabolism under respiration-permissive conditions (see equation 3). However, under respiration-permissive conditions, NADH can donate electrons to the ETC, with O_2_ being the final acceptor. The estimated NADH reoxidized in the ETC due to O_2_ uptake (see equation 4) showed that the overall NADH-NAD^+^ balance was close to zero also under respiration-permissive conditions (see equation 5; Table 2). Acetoin production was associated with 71% ± 13% of the NADH consumed in respiration.

**TABLE 2:** Overall NADH-NAD^+^ balance (*Y*_SNADH_) and substrate-level phosphorylated ATP (*ATP*_SLP_) estimated from metabolite production per mol of consumed lactose under anaerobic (AN) and respiration-permissive (RES) conditions^*a*^

Dilution rate (h^−1^)	*Y*_SNADH, catabolism_	*Y*_SNADH, ETC_	*Y*_SNADH_	Estimated *ATP*_SLP_
	(mol mol^−1^) (equation 2/equation 3)	(mol mol^−1^) (equation 4)	(mol mol^−1^) (equation 5)	(mol mol^−1^) (equation 6)
AN 0.1 h^−1^	0.16 ± 0.02	NA	0.16 ± 0.02	4.28 ± 0.1
AN 0.5 h^−1^	0.08 ± 0.02	NA	0.08 ± 0.02	4.27 ± 0.6
AN 0.8 h^−1^	0.03 ± 0.02	NA	0.03 ± 0.02	3.97 ± 0.4
RES 0.1 h^−1^	3.34 ± 0.17	3.56 ± 0.58	–0.22 ± 0.67	3.36 ± 0.16
RES 0.5 h^−1^	3.00 ± 0.18	2.75 ± 0.11	0.25 ± 0.18	3.87 ± 0.23
RES 0.8 h^−1^	2.34 ± 0.23	2.51 ± 0.20	–0.17 ± 0.11	3.28 ± 0.26

a The NADH-NAD^+^ balance is expressed as the net yield of produced NADH on consumed substrate *S* (*Y*_SNADH_). Values given are the average of biological duplicates and the 95% confidence interval from measurements of two separate steady-state samples from each cultivation. NA, not applicable.

The estimated ATP formed by substrate-level phosphorylation (*ATP*_SLP_; see equation 6) was lower under respiration-permissive conditions at all dilution rates (*P* < 0.05), despite the fact that the biomass yields were significantly higher (Table 2).

### Degree of cell damage at each process step.

**FIG 5:**
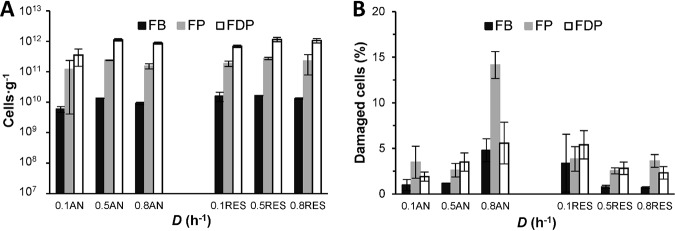
Flow cytometry of cells in the fermentation broth (FB, black), frozen product (FP, gray), and freeze-dried product (FDP, white). (A) Total number of cells; (B) percentage of damaged cells of L. lactis CHCC2862 in each process step and dilution rate after harvesting from anaerobic (AN) and respiration-permissive (RES) chemostats. The error bars represent the 95% confidence interval from measurements of two biological replicates.

Cell robustness is essential throughout the production process to ensure starter cultures of high quality. To investigate the effect of fermentation conditions on cellular robustness, cells were harvested from each fermentation experiment after steady-state samples had been collected. The complete fermentation broth (FB) was divided into two equal parts that were used to produce frozen product (FP) and freeze-dried product (FDP), respectively (Fig. 2). The robustness was assessed by measuring the number of cells and their membrane potential using flow cytometry to estimate the percentage of damaged cells after each process step (Fig. 5). The total number of cells per gram increased between the downstream process steps as cells were concentrated by centrifugation and freeze-drying (Fig. 5A). According to cell counts measured in each of the process steps (FB, FP, FDP), cell recoveries after downstream treatments were complete within 3% ± 2% and 7% ± 1% for FP and FDP, respectively. In general, cells in the frozen and freeze-dried products had a higher degree of cell damage than those in the fermentation broth (Fig. 5B). There was a tendency toward an increasing percentage of damaged cells with increasing dilution rate under anaerobic conditions, and the highest percentage of damaged cells (14%) was determined in the frozen product harvested at *D* = 0.8 h^−1^. Cells harvested under respiration-permissive culture conditions showed the opposite trend; the percentage of damaged cells decreased with increasing dilution rate and was only about 3% in the frozen and freeze-dried products harvested at 0.5 h^−1^ and 0.8 h^−1^, respectively (Fig. 5B).

### Acidification performance.

**FIG 6:**
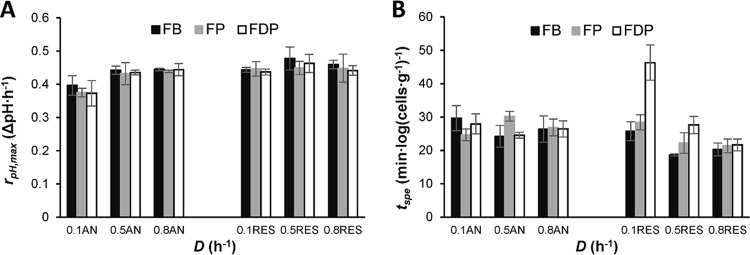
Acidification activity of cells in the fermentation broth (FB), frozen product (FP), and freeze-dried product (FDP) after cultivation under anaerobic (AN) and respiration-permissive (RES) conditions. (A) Maximum acidification (*r*_pH,max_). (B) Specific acidification time (*t*_spe_). The error bars represent the 95% confidence interval from measurements of two biological replicates.

The effect of the cultivation conditions on the acidification performance after each process step was assessed by a standardized milk acidification assay (Fig. 6). For anaerobically cultured cells, the downstream processing alone did not seem to influence the maximum acidification rate (*r*_pH,max_) between cells in the fermentation broth (FB), frozen product (FP), and freeze-dried product (FDP). However, it was significantly affected by low dilution rates in all process steps. At *D* = 0.1 h^−1^, the maximum acidification rates of FB, FP, and FDP were about 13% lower than at the higher dilution rates (Fig. 6A; *P* < 0.05). No significant differences were found in the specific acidification time (*t*_spe_) across product types or the dilution rates, although the freeze-dried products tended to perform slightly better at the higher dilution rates (Fig. 6B).

In respiration-permissive conditions, the maximum acidification rate (*r*_pH,max_) appeared to be independent of the dilution rate, and no significant differences were observed among the fermentation broth and the frozen and freeze-dried products prepared (Fig. 6A). On the other hand, the specific acidification time (*t*_spe_) depended on both the dilution rate and the downstream processing. After cultivation at the two higher dilution rates, the *t*_spe_ for the FB, FP, and FDP products was shorter (i.e., better) than at *D* = 0.1 h^−1^ (Fig. 6B). At low dilution rates, the cells were highly sensitive to freeze-drying. The specific acidification time of the freeze-dried product was more than 60% longer than that of the frozen product at *D* = 0.1 h^−1^. The sensitivity to freeze-drying was less severe at the higher dilution rates, although a difference in *t*_spe_ could still be observed between the freeze-dried and the frozen products at *D* = 0.5 h^−1^. Moreover, compared to the anaerobic conditions, the frozen products of *D* = 0.5 h^−1^ and 0.8 h^−1^ and the freeze-dried product of *D* = 0.8 h^−1^ resulted in similar *r*_pH,max_ and shorter *t*_spe_ than their anaerobic counterparts, making the respiro-fermentative cells at *D* = 0.5 h^−1^ and 0.8 h^−1^ the best-performing products among all conditions tested.

## DISCUSSION

### Metabolic shifts and respiration in Lactococcus lactis.

The metabolic shift between homofermentative and mixed-acid fermentation under anaerobic conditions can be strain dependent, although factors such as carbon excess, high glycolytic flux, and rapid growth are often associated with homofermentative metabolism. Mixed-acid fermentation, on the other hand, is associated with low specific growth rate, carbon limitation, and low glycolytic flux (11, 14). Aerobic conditions appear to lead to improved growth yields in most lactic acid bacteria (20). However, while six out of six strains of L. lactis displayed a respiration-associated metabolic shift upon aeration and addition of heme (21), only about 20% of 76 screened strains of *Lactobacillus*, *Leuconostoc*, and *Weissella* species showed respiratory phenotypes (20).

We used chemostat cultures under anaerobic and respiration-permissive conditions to enable quantitative analysis of the metabolic shifts between homofermentative fermentation, mixed-acid fermentation, flavor formation, and respiration in L. lactis CHCC2862 using an industry-like rich medium. We found that L. lactis CHCC2862 remained homofermentative in anaerobic chemostats, regardless of the dilution rate. The acetate yield on lactose increased with decreasing dilution rates, but the overall yield of lactate on consumed lactose was above 96% in all anaerobic chemostats.

In contrast, under respiration-permissive conditions the metabolite production profile varied with the dilution rate. In conjunction with respiration, the flavor-forming and mixed-acid pathways were active in L. lactis CHCC2862 at all growth rates, but clear differences were observed in lactate formation. The lactate concentration was below the HPLC detection limit at the lowest dilution rate, while a mixture of lactate and acetoin, acetate, and CO_2_ was produced at the two higher dilution rates. Several enzymes, primarily LDH, ALS, PDH, and PFL, compete for pyruvate as the substrate, and LDH also competes with, e.g., NADH oxidase for NADH. It was clear from the overall yields that, at the highest dilution rates, the most active enzymes were ALS and LDH, as the pyruvate flux was directed toward acetoin and lactate. Acetate was also formed, but the specific rate of acetate formation plateaued, along with decreasing yield of acetate on lactose. This may be attributed to allosteric regulation of several enzymes by fructose-1,6-bisphosphate (FBP). The FBP level can be expected to increase at high specific growth rates (13, 14, 22). FBP increases the affinity of LDH toward both pyruvate and NADH (23), and it is known to inhibit PTA and ACKA, which are involved in acetate formation (22, 24). Together, this would mean that carbon flux is redirected away from acetate toward lactate at high dilution rates. It is, however, interesting that such a shift was not clearly seen under anaerobic conditions in this strain, which showed similar yields at all anaerobic steady states. Tentatively, this could be due to regulation via the NADH/NAD^+^ ratio, which should generally be higher under anaerobic than under aerobic conditions. Moreover, at *D* = 0.1 h^−1^, the specific lactose uptake rate, which translates into the glycolytic rate, was almost three times higher under anaerobic conditions (*q*_S_ = 3.25 ± 0.41 mmol gDW^−1^ h^−1^) than under respiration-permissive conditions at the same dilution rate (*q*_S_ = 1.22 ± 0.09 mmol gDW^−1^ h^−1^).

Previous studies have shown that few changes in transcription and protein synthesis are direct results of respiration-permissive conditions (7, 25). It has been suggested that NADH is a key regulator in carbon metabolism, as a low NADH/NAD^+^ ratio could allosterically, or via substrate concentration-related effects, redirect metabolism in favor of enzymes using NAD^+^ or pyruvate as the substrate, without necessarily changing the gene expression levels or enzyme concentrations (12, 26). Recently, Cesselin and colleagues analyzed the transcriptional response in early stationary phase under respiration-permissive conditions and found that late changes in the transcriptome included significant upregulation of several genes in the acetate (*pdhABCD*, *poxL*, and *pflB*) and acetoin (*als*, *aldB*, *butA*, and *butB*) pathways (21). Using mutants with inactivated *pdhA*, *pfl*, *pta*, and *als*, they concluded that acetate production is necessary for the increased biomass yield, while the acetoin production, being pH neutral, alleviates acid stress and prolongs survival. Moreover, pyruvate accumulation was identified as the main driver for *als* activity (21). The kinetic properties of ALS, PDH, and LDH also favor acetoin production at high intracellular pyruvate concentrations (27).

Our results suggest that, in addition, acetoin production is required to compensate for the NADH consumed in respiration. We calculated that there was a net production of NADH from catabolism of lactose in respiratory cultures. This is related to the higher levels of flavor-forming and mixed-acid products. Under respiration-permissive conditions at the lowest dilution rate, almost no lactate was produced, and acetoin and CO_2_ were produced instead. If no lactate is formed, NADH must be oxidized either via ethanol formation or via the ETC. Only traces of ethanol were observed, regardless of the dilution rate, which leads us to the conclusion that NADH is oxidized via the ETC. We measured the oxygen uptake rate and found that the respiratory NADH consumption aligned with the net catabolic NADH production to within ±8%, indicating that the net NADH production in catabolism is well balanced with the NADH consumption due to respiration.

The increase in biomass yield is a clear result of heme-activated respiration, whether additional energy is released or whether the energy demand is reduced. Under anaerobic conditions, the H^+^-ATPase transfers protons across the cytoplasmic membrane at the expense of ATP to maintain the intracellular pH (8, 16). Under respiration-permissive conditions, the flow of electrons through the ETC extrudes protons without any additional need for ATP, thereby reducing or eliminating the cost of cellular ATP proton transfer (8). However, respiration in L. lactis is different from that in other respiring organisms such as E. coli and B. subtilis, which use the Krebs cycle to produce NADH for full respiration. L. lactis does not possess all the enzymes required for a complete Krebs cycle and thus generates NADH via fermentation. During respiration, the reoxidation of NADH takes place via the ETC, and the accumulated pyruvate must therefore be consumed via NADH-producing or redox-neutral reactions, such as acetoin formation, to support respiration, which is completely in line with our results and previously reported data (21).

### Cellular economy.

Respiration capacity is often related to both the NADH/NAD^+^ and the ATP/ADP ratios and affects biomass production. However, NADH oxidation and ATP production are only weakly connected in L. lactis, as most of the ATP is generated by substrate-level phosphorylation (15). The function of H^+^-ATPase in relation to the ETC has not yet been fully elucidated, and the question is whether H^+^-ATPase contributes with additional ATP besides that produced in glycolysis and mixed-acid fermentation (15). We estimated the substrate-level phosphorylated ATP (*ATP*_SLP_) from the metabolite yields (Table 2). Anaerobic conditions led to higher *ATP*_SLP_ values than respiration-permissive conditions did. Nevertheless, the biomass yield was significantly increased under all respiration-permissive conditions studied. At the lowest dilution rate, the biomass yield increased 165% compared to anaerobic conditions, whereas it increased 36% and 23% at *D* = 0.5 h^−1^ and *D* = 0.8 h^−1^, respectively (Table 1).

Heme-activated respiration indisputably increases biomass yield. However, it is not clear if this is because additional ATP is formed or because the cellular ATP requirement is reduced. When environmental conditions are favorable and nutrient supplies are abundant, microorganisms direct nutrient and energy resources primarily toward growth-related cellular processes (28, 29). In contrast, when nutrient and energy supplies are limited, microorganisms predominantly employ metabolic energy for maintenance-related processes, which can lead to shifts in metabolic pathways, including changes in energy storage and utilization pathways (30). The maintenance coefficient obtained under anaerobic conditions (Fig. 4A) corresponds to previously reported values of comparable L. lactis strains, such as L. lactis subsp. *lactis* IL-1403 (31, 32). The maintenance energy demand is the likely reason for the lower biomass yield at the lowest dilution rate under both anaerobic and respiration-permissive conditions (Table 1).

The *m*_S_ obtained from respiration-permissive conditions was only 10% of the anaerobic value. This explains the large difference between anaerobic and aerobic biomass yields at *D* = 0.1 h^−1^, as a larger fraction of the lactose consumption would be used for maintenance energy requirements under anaerobic conditions, instead of providing energy for biomass formation. In addition, when excluding the biomass formation, the carbon recovery was below 90% for all the respiratory cultures and around 100% for the anaerobic cultures. Since the calculated carbon recoveries did not include carbon potentially incorporated into biomass, the low carbon recoveries suggest that lactose was assimilated into biomass under respiration-permissive conditions.

These observations indicate either that additional ATP formation could take place from the available lactose due to respiration or that respiration-permissive conditions cause a lower energy requirement for maintenance. Our data do not allow us to clearly distinguish between these mechanisms, but both would lead to fulfilment of the maintenance energy demand at a lower substrate consumption rate.

### Cellular robustness.

In industry, cells are usually cultivated in batch fermentation and harvested in the stationary phase. Batch cultivations are characterized by dynamic changes throughout the process. Early in the batch, the substrate concentration and the specific growth rate are high. Toward the end, the substrate is depleted, product concentrations accumulate, and the specific growth rate decreases. This activates various stress resistance mechanisms due to nutrient limitations, leading to an increased tolerance to other stress conditions, such as freezing and freeze-drying (1). Previous studies have shown that fermentation conditions and harvest time influence the cellular robustness of starter cultures (3). In the present study, cells were instead harvested at steady state in chemostat cultures to investigate how the physiological state of cells affects frozen and freeze-dried products. At steady state, growth occurs at a constant rate lower than the maximum specific growth rate and all culture variables remain constant. This offers the possibility to examine the cell biology in a quantitative and controlled manner. Our results showed that at high dilution rates, a higher degree of cell damage was observed under anaerobic conditions than under respiration-permissive conditions, indicating that respiration leads to more robust cells as long as it occurs in parallel with lactate production at an intermediate or high specific growth rate. The reasons for this are not clear, but the lower acid stress and modulation of the membrane composition in the presence of oxygen may affect the robustness of the final product (21, 33).

The acidification activity is an important technological property of industrial dairy starter cultures. The adaptation of cells to a milk medium after their production is crucial for successful acidification in standard dairy production processes. Under anaerobic conditions, the initial acidification activity, here expressed as the specific acidification time (*t*_spe_), did not differ significantly between the three types of product formulations (fermentation broth, frozen, and freeze-dried). However, in respiration-permissive conditions, freeze-dried products had a slightly longer specific acidification time at *D* = 0.5 h^−1^, and much longer at *D* = 0.1 h^−1^, than at *D* = 0.8 h^−1^. These observations suggest that the metabolic shift toward flavor-forming pathways affects the initial ability of freeze-dried cells to acidify milk. They also highlight that the freeze-drying process may have a huge impact and should be included in investigations of the robustness of starter cultures.

The maximum acidification rate (*r*_pH,max_) was not significantly affected by any of the cultivation conditions, except by anaerobic growth at *D* = 0.1 h^−1^, after which it was on average about 13% lower than in the other cases (*P* < 0.05). The reason for the low value of *r*_pH,max_ at low specific growth rates under anaerobic conditions may potentially be the high-maintenance energy demand, hypothetically leading to loss of function resulting from energy limitation. The relatively high values of both *r*_pH,max_ and *t*_spe_ in the freeze-dried product prepared after respiration-permissive cultivation at *D* = 0.1 h^−1^ indicate that the shift of metabolism does not change the potential metabolic acidification rate but does affect the ability of the cells to adapt and initiate growth and milk acidification. The physiological state of cells under respiration-permissive conditions at *D* = 0.1 h^−1^ suppresses lactate formation, which is the principal acidification activity. Furthermore, in respiration-permissive conditions at *D* = 0.1 h^−1^, high levels of acetoin were present which may have contributed to the poor adaptation, since acetoin has been shown to inhibit cell growth (34).

L. lactis starter cultures are produced in batch cultivations striving for high biomass yield of robust high-performance cells. Chemostat cultivations represent snapshots of what takes place at different points in a batch cultivation. Clearly, the respiratory metabolism was quite flexible. Low specific growth rate resulted in undetectable lactate formation and high acetoin formation. The frozen products of *D* = 0.5 h^−1^ and 0.8 h^−1^ and the freeze-dried product of *D* = 0.8 h^−1^ exhibited the highest acidification activity among all tested conditions, whereas cells produced under respiration-permissive conditions at the lowest dilution rate performed very poorly after freeze-drying. This raises the question of whether the activity of the LDH pathway is beneficial or if the high level of acetoin is detrimental for cellular robustness during storage of starter cultures. The harvest point in current starter culture production setup takes place in the stationary phase, where the specific growth rate is very low. To achieve high L. lactis biomass yield and high-performance freeze-dried starter cultures, one may want to reconsider this harvesting procedure.

In conclusion, this study provides a quantitative analysis of chemostat cultures of L. lactis under respiration-permissive and anaerobic conditions. Respiration-permissive conditions gave clearly higher biomass yield, ranging from 0.29 to 0.33 C-mol C-mol^−1^, than anaerobic conditions, 0.11 to 0.26 C-mol C-mol^−1^. Lactate formation was eliminated under respiration-permissive conditions at a dilution rate of *D* = 0.1 h^−1^. Respiratory cultures have very low maintenance requirements compared to anaerobic cultures, which is probably the main reason for the increased biomass yield. Respiration-permissive conditions have been reported to have several advantages in previous studies, and our findings confirm their superior performance at high dilution rates but not necessarily in freeze-dried products obtained at lower dilution rates. This work thus underlines the importance of systematically studying the upstream as well as the downstream aspects of production processes.

## MATERIALS AND METHODS

### Upstream procedure.

**(i) Organism and preculture preparation.** The strain used in this study was Lactococcus lactis subsp. *lactis* CHCC2862, an industrial strain used in cheese-making (Chr. Hansen Culture Collection, Hørsholm, Denmark). Strain material was transformed into glycerol stocks using overnight static cultivation in M17 medium (Oxoid) and 5 g liter^−1^ lactose (Merck). The working volume was 100 ml, the temperature was 30°C, and the starting pH was 6.5. At harvest, the broth was mixed with glycerol solution to a final volume ratio of 15% (vol/vol), divided into vials of 1.0 ml, and stored at –50°C. For each cultivation condition, a new glycerol stock vial was used to inoculate static precultures containing M17 medium and 5 g liter^−1^ lactose in a working volume of 100 ml, at a temperature of 30°C and starting pH of 6.5. Cells were harvested in the late growth phase before lactose depletion and added to the bioreactor to achieve an initial optical density at 600 nm (OD_600_) of 0.06.

**(ii) Fermentation procedure.** The fermentation medium was an M17-based medium modified to support high sugar concentrations, consisting of the following (g liter^−1^): yeast extract, 7.5 (Oxoid); MgSO_4_ · 7H_2_O, 0.75 (Merck); tryptone, 15 (BD); soya peptone, 15 (Oxoid); lab Lemco powder, 15 (Oxoid); ascorbic acid, 0.7 (VWR); and lactose, 40 (Merck) (36). Hemin stock solution was added to a final concentration of 10 mg liter^−1^ to obtain respiration-permissive conditions. Hemin (Sigma) stock solution (0.5 g liter^−1^), was prepared in alkaline water (containing 0.05 M NaOH) and sterile filtered with a 0.8/0.2-μm Supor membrane filter. A fresh stock solution was prepared before every fermentation experiment. It should be noted that heme and hemin correspond to ferrous (reduced) and ferric (oxidized) iron in the protoporphyrin ring, respectively. In this work we refer to “heme” regardless of the iron redox state.

All fermentations were carried out in a 1.5-liter Sartorius Biostat B twin tower with a 1-liter working volume. Initially, 0.5 ml liter^−1^ antifoam (suppressor 3130; Hydrite) was added. The temperature was 30°C and the pH set point was 6.0, adjusted with 24% ammonium hydroxide. Anaerobic conditions were established by continuously flushing the headspace with nitrogen gas at 0.1 liter min^−1^ at an agitation rate of 300 rpm. Under respiration-permissive conditions, the bioreactors were sparged with air. A minimum threshold of 60% dissolved oxygen was ensured by using cascade control, i.e., gradually increasing the stirrer speed and the aeration rate using Sartorius control software. The initial airflow was set to 0.5 liter min^−1^, and the agitation rate was 400 rpm. The outlet gas was monitored with a Prima BT benchtop mass spectrometer (Thermo Fischer Scientific).

All chemostat fermentations were initially run in batch mode. Batch operation was switched to chemostat mode at the late exponential growth phase before lactose depletion, using the same cultivation conditions. We aimed to achieve the three dilution rates, 0.1 h^−1^, 0.5 h^−1^, and 0.8 h^−1^, for both anaerobic and respiration-permissive conditions. However, the actual dilution rates were 0.11 h^−1^, 0.52 h^−1^, 0.74 h^−1^, and 0.78 h^−1^ for anaerobic conditions. For respiration-permissive conditions, the actual dilution rates were 0.11 h^−1^, 0.52 h^−1^, and 0.8 h^−1^. Steady states were verified after a minimum of eight residence times by repeated measurements of CO_2_, OD_600_, and metabolites. All fermentations were performed in biological duplicates, and each steady state originated from an independent preculture and chemostat startup. The results given are based on two measurements at each steady state and each biological replicate, separated by at least one residence time.

**(iii) Sampling.** Steady-state sampling was performed by withdrawing approximately 15 ml broth and transferring it directly to a 50-ml Nunc tube containing stainless steel beads (40 g, 4 mm diameter) precooled to –30°C. The sample did not freeze and was quickly distributed to more analysis tubes (37).

### Downstream procedure: preparation of frozen and freeze-dried product.

Fig. 2 illustrates the downstream process steps performed in this study. Cells were harvested from each fermentation experiment after steady-state samples had been collected. The broth was divided into two equal parts; one was used to produce the frozen product (FP), and the other, the freeze-dried product (FDP). Both were concentrated using centrifugation (Heraeus Multifuge X3 FR; Thermo Fisher Scientific) at 4,000 rpm for 20 min at 4°C. The supernatant was discarded, and the concentrate was resuspended. A cryo-additive was added to the cells intended for freeze-drying at a ratio of 1:1 (1 g dry matter of concentrated cells per 1 g dry matter of cryo-additive) prior to freezing. The cryo-additive contained 6% skim milk, 8% trehalose, and 4% sodium ascorbate and was sterilized at 110°C for 20 min.

The resuspended concentrates (with or without cryo-additive) were pelletized by dropwise freezing in liquid nitrogen using a 25-ml single-use pipette. The frozen products (FP) were subsequently stored at –50°C until analysis or freeze-drying (FDP).

Freeze-drying of a known weight of frozen pellets in preweighed containers was performed on shelves precooled to –50°C in a Telstar LyoBeta 15 freeze-dryer. After a holding step of 8 to 9 h at –50°C, the chamber pressure was decreased to 30 pascals, and the shelf temperature was increased from –50 to 30°C to initiate sublimation. The drying process continued until there was no further weight loss of the product, after which, the vacuum was released by injecting air. FDP samples were packed in aluminum foil packaging bags, sealed, and stored at –50°C until analysis.

### Analytical methods.

**(i) Biomass measurements.** Optical density was measured at 600 nm (OD_600_) with a Hitachi U-1900 spectrophotometer after appropriate dilution with 0.9% NaCl. The dry weight of the fermentation samples was measured gravimetrically using 0.2 μm polyethersulfone membrane filters. The correlation between OD_600_ and cell dry weight (DW) was estimated to be 0.35 OD gDW^−1^. The moles of carbon (C-mol) of biomass was calculated by assuming the same biomass composition under all fermentation conditions and using the biomass composition of L. lactis grown in M17, CH_1.70_O_0.51_N_0.22_ (38).

**(ii) Substrate and metabolite analysis.** *(a) Lactose and lactate.* Samples were prepared by adding 200 μl 4 M sulfuric acid (H_2_SO_4_) to a 1-ml sample to arrest metabolic activity. Prior to analysis, 1.8 ml perchloric acid and 2.0 ml internal standard solution were added, followed by centrifugation for 30 min at 4°C and 2,800 × *g*. Final dilutions were made using Milli-Q water to ensure that the concentration was within the analysis limits/range of the equipment. Measurements of lactose and organic acids were performed using an ICS-3000 ion chromatography dual system. Lactose was analyzed with a CarboPac PA-20 3 × 150-mm column using an eluent gradient of KOH in Milli-Q water. The initial concentration was 0.19 mM KOH, increasing linearly to 100 mM KOH at 10.5 to 19 min and then decreasing instantaneously back to 0.19 mM at 27 to 27.5 min. The flow was 0.4 ml min^−1^ with a start pressure of 2,500 to 2,900 lb/in^2^, and the column temperature was set to 25°C. Lactate was analyzed with an IonPac ICE As6 9 × 250-mm column using 0.4 M heptafluorobutyric acid as the eluent. The flow was 1.0 ml min^−1^, the start pressure was 750 to 900 lb/in^2^, and the column temperature was set to 26°C.

*(b) Acetoin and ethanol.* Acetoin and ethanol were measured using headspace gas chromatography with a Perkin Elmer flame ionization detector. Samples were prepared by transferring 1 ml fermentation broth directly into a headspace vial containing 200 μl 4 M H_2_SO_4_ to stop metabolic activity, after which the vial was sealed with a crimped Teflon-lined aluminum cap. The headspace vials were heated to 70°C for 36.5 min prior to analysis. The gas was analyzed using a polar 25 m × 0.2 mm × 0.33 μm HP-FFAP column (Agilent Technologies). The oven temperature was held at 80°C for 2 min and then ramped to 230°C and held for 0.5 min.

*(c) Acetate and formate.* The analysis of acetate and formate was like that of acetoin and ethanol, although sample preparation differed as follows: 1 ml of fermentation broth was transferred to a headspace vial containing 1 ml saturated sodium bisulfate solution, and 1 ml 96% ethanol was added before the vial was sealed with a Teflon-lined aluminum cap. Ethanol reacts with acetate and formate, forming ethyl esters with a lower boiling point, which were analyzed using headspace gas chromatography as describe above.

**(iii) Acidification activity.** The acidification activity was measured using the Cinac system (Alliance Instruments, France). The method measures the decrease in pH and estimates the acidification activity and how fast cells adapt to the applied conditions. The acidification assay was carried out in bottles containing 200 ml standardized milk (autoclaved skim milk powder and water with a dry matter content of 9.5%) at 30°C for 16 h. The bottles were inoculated with the selected culture product at the following different inoculation percentages (wt/wt): 0.1% for fermentation broth samples, 0.02%, 0.01%, and 0.005% for frozen products, and 0.005%, 0.0025%, and 0.00125% for freeze-dried products. Before starting the pH measurements, equilibrium was ensured between the pH of the milk and the electrode. The pH was continuously monitored in the inoculated milk and led to the determination of the maximum acidification rate, *r*_pH,max_ (ΔpH h^−1^) and the time necessary to obtain a decrease of 0.08 pH units, *T*_0.08_ (min). The higher the *T*_0.08_, the longer the latency phase and the lower the acidification activity. The specific acidification time, *t*_spe_ (min log[cells g^−1^]^−1^) was defined as the ratio of *T*_0.08_ to the corresponding log of cell concentration and was calculated according to equation 1 (39).

(1) $$t_{\mathrm{spe}}=\frac{T_{0.08}}{\text{log}(\mathrm{cells}g^{-1})}$$

**(iv) Membrane potential.** Flow cytometry was used to analyze cell counts and determine the ability of cells to maintain a membrane potential. Prior to analysis, cells were reactivated in MRS medium for 30 min at 40°C. The cells were then added to a stain mix, the temperature was lowered to 20°C, and the mixture was allowed to stand for 30 min. The stain mix contained 210 μl 50% (wt/wt) glucose solution, 210 μl 1.5 mM 3,3′-diethyloxacarbocyanine iodide (DiOC 3.3′) solution in dimethyl sulfoxide, and 50 g bead mix, which was used as an internal standard in the flow cytometry. After staining, the samples were analyzed with a BD LSRFortessa flow cytometer using a blue laser at 488 nm. The conversion of sugar to lactate produces protons inside the cell which are actively pumped into the environment. This creates an electric gradient over the cell membrane, which drives the diffusion of DiOC 3.3′ across the cell membrane into the intracellular space, resulting in red fluorescence. The increase in intensity of the red fluorescence provides a measure of the membrane potential and was detected using the far-red parameter. The far-red parameter differentiates cells into two separated groups, those that are able to create a membrane potential (intact cells) and those that are not (damaged cells).

**(v) Calculation of energy parameters.** *(a) Substrate-related maintenance.* The substrate-related maintenance coefficient (*m*_S_) and maximum biomass yield on lactose (*Y*_SX_^max^) were predicted by linear regression of the specific substrate consumption rate (*q*_S_) versus the dilution rate (*D*) (35). The slope represents 1/*Y*_SX_^max^, and the intercept on the *y* axis gives *m*_S_.

*(b) NADH-NAD^+^ balance.* The overall NADH-NAD^+^ balance was evaluated from the metabolite yields according to equations 2 to 5, where *Y*_Si_ is the yield of metabolite *i* on consumed lactose (*S*). Under anaerobic conditions, it was assumed that acetate was formed via PFL. The overall net NADH formation in catabolism (*Y*_SNADH, catabolism_) was therefore calculated according to equation 2.

(2) YSNADH, catabolism=YSAcetate–YSEthanol

Under respiration-permissive conditions, it was instead assumed that acetate was formed via PDH, as no formate was detected. Furthermore, NADH consumption in the ETC was estimated from the oxygen uptake. The net NADH formation in catabolism (*Y*_SNADH, catabolism_) and net NADH consumption due to respiration (*Y*_SNADH, ETC_) were therefore calculated according to equations 3 and 4,

(3) $$Y_{\text{SNADH},\text{ catabolism}}={\text{ 2}Y}_{\text{SAcetate}}+{\text{ 2}Y}_{\text{SAcetoin}}+{\text{ 2}Y}_{\text{SDiacetyl}}–Y_{\text{SEthanol}}$$

(4) $$Y_{\text{SNADH},\text{ ETC}}=\text{ 2}Y_{\text{SOxygen}}–Y_{\text{SDiacetyl}}$$

The overall net NADH formation was calculated as the difference between the NADH formed in catabolism and consumed in the ETC,

(5) $$Y_{\text{SNADH}}=Y_{\text{SNADH},\text{ catabolism}}–Y_{\text{SNADH},\text{ ETC}}$$

the second term being zero under anaerobic conditions.

*(c) Substrate-related ATP yield.* The net amount of ATP produced by substrate-level phosphorylation per consumed lactose, *ATP*_SLP_, was calculated from the product yields according to equation 6.

(6) $$\mathrm{AT}P_{\text{SLP}}=Y_{\text{SLactate}}+\text{ 2}Y_{\text{SAcetate}}+Y_{\text{SEthanol}}+\text{ 2}Y_{\text{SAcetoin}}+\text{ 2}Y_{\text{SDiacetyl}}$$

**(vi) Data analysis.** The impact of each cultivation condition and product performance was statistically analyzed using the two-sample *t* test, where statistical differences between means were tested at a 5% level of significance. Where applicable, values are reported as 95% confidence intervals (average ± SD t_0.025, df_).

The significance of regressions of the linear rate equations, used for estimation of the maintenance coefficients, was assessed using analysis of variance (ANOVA), more specifically the *F* test, by calculating the test statistic *F*_obs_, while the 95% confidence intervals of individual parameters were estimated using the *t* test.
